# From Inconclusive Fine Needle Aspiration to Double Malignancies: A Case Report of Primary Thyroid Lymphoma With Concurrent Papillary Thyroid Carcinoma in a Sickle Cell Disease Patient in Saudi Arabia

**DOI:** 10.7759/cureus.90713

**Published:** 2025-08-22

**Authors:** Majid H Ali, Waref H Felemban, Rawan E Hudairy, Afnan A Alnamankany, Mazen M Alkazmi, Alhasan A Bajawi

**Affiliations:** 1 Department of Medicine, Al-Noor Specialist Hospital, Makkah, SAU; 2 Department of Medicine, Umm Al-Qura University, Makkah, SAU; 3 Department of Histopathology, Al-Noor Specialist Hospital, Makkah, SAU

**Keywords:** inconclusive fna, papillary thyroid microcarcinoma, pet ct, primary thyroid lymphoma, sickle cell disease

## Abstract

Primary thyroid lymphoma (PTL) is considered a rare and aggressive form of malignancy. Reaching a diagnosis might be challenging due to the diverse clinical and histopathological presentation and inconclusive cytology, especially in patients diagnosed with Hashimoto's thyroiditis, as results may be affected by the chronic inflammatory changes. The simultaneous existence of PTL with papillary thyroid microcarcinoma is relatively rare, as indicated by the current literature and reported cases worldwide and locally. A 45-year-old woman with hypothyroidism and sickle cell disease (SCD) presented initially with an incidental nodule found on routine ultrasound. Initial fine needle aspiration (FNA) was suggestive of Hashimoto's thyroiditis, and the patient underwent serial monitoring in the outpatient department (OPD), which subsequently revealed an extensively growing neck mass. Due to rapid evolution and increasing mass symptoms, the endocrine surgery team performed a total thyroid gland removal. Post-resection thyroid tissue histopathology revealed the presence of high-grade B-cell lymphoma involving both lobes and an incidental occurrence of papillary thyroid carcinoma (PTC). The patient tolerated chemotherapy with continued surveillance and observation. Post-chemotherapy computed tomography positron emission tomography (PET-CT) scan demonstrated a new lesion with increased uptake of F-fluorodeoxyglucose (FDG) near the thyroid, following postoperative anatomical changes, which initially appeared representative of relapse. Nonetheless, a surgical biopsy demonstrated benign lymphoid hyperplasia, and the patient remains in remission. This case exemplifies the potential limitations of FNA in PTL, the diagnostic challenge of double malignancies, and the importance of histological validation for PET-positive lesions in continuous OPD follow-up and surveillance.

## Introduction

Thyroid malignancies comprise 1% of malignancies. Thyroid malignancies, predominantly present as painless or painful neck mass with possible associated compressive symptoms such as dysphagia or dyspnea, depending on the degree and the extent of the mass, are classified mainly on the histopathological findings, immunohistochemistry, and molecular testing. Papillary thyroid carcinoma (PTC) is the most prevalent, with more than two-thirds of the population of thyroid cancers, followed by follicular type, medullary thyroid cancer, and, lastly, the least common anaplastic thyroid cancer. Early identification and initiation of therapeutic goals play a vital role in the prognosis. Primary thyroid lymphoma (PTL) is a rare subtype of thyroid malignancy, with a prevalence of approximately 1-5% of all thyroid cancers and 2% of extranodal lymphomas. It most frequently emerges in the context of established chronic autoimmune thyroiditis, specifically Hashimoto's thyroiditis, which raises the risk of lymphoid proliferation in the thyroid gland. Diffuse large B-cell lymphoma (DLBCL) is reported to be the most common histological subtype of PTL and is commonly aggressive [1,2].

The diagnosis begins by acknowledging the vast clinical nature of thyroid malignancy, addressing the possibility of cytological overlap between malignant findings and chronic Hashimoto's thyroiditis, which may result in inaccurate histopathological assessment. A recent retrospective observational study highlighted the limited role of fine needle aspiration (FNA) in finalizing the diagnosis of PTL and concluded that core or surgical biopsy is the appropriate diagnostic modality. While core needle biopsy offers an improved diagnostic yield, careful histopathological assessment and examination are warranted in the context of rapid clinical progression or compressive symptoms [2].

The simultaneousness of PTL and PTC is particularly rare, with merely a periodic reported cases in the literature [3,4]. In patients with systemic inflammatory conditions, such as sickle cell disease (SCD), diagnostic performance is further complicated due to the activated baseline immune system and changed tissue reactions, medication history, and its interference with the overall disease course [5].

In Saudi Arabia, the prevalence of PTL is incredibly lower compared to other forms of malignancies. A two-decade review of thyroid malignancies incidence in major advanced tertiary care sectors shows that papillary carcinoma substantially dominates thyroid cancers, with lymphomas making up a small percentage (<1%) [6].

In the present case, we report a patient with SCD who presented with high-grade B-cell thyroid lymphoma and concurrent spontaneous papillary thyroid microcarcinoma. For diagnosis, a complete histopathological assessment is crucial, highlighting critical diagnostic pitfalls and emphasizing the importance of tissue confirmation in the diagnosis of thyroid malignancies.

## Case presentation

**Figure 1:**
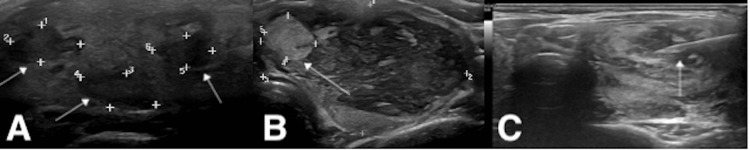
First ultrasound of the thyroid (A) Multiple variable-sized nodules (largest in the RT lobe measuring 0.66 x 1.2 cm; RT lobe hypoechoic nodule = Thyroid Imaging Reporting and Data System (TIRADS) IV; RT lobe with peripheral calcification = TIRADS IV-V). (B) The LT lobe was occupied by a large, solid, heterogeneous nodule (large LT lobe nodule = TIRADS II; hyperechoic module = TIRADS III). Doppler study showed increased gland vascularity. (C) Under aseptic precautions and local anesthesia, a fine needle aspiration (FNA) was carried out from the left thyroid nodule using a 23-G needle. FNA and cell block were obtained. The specimen was inspected immediately by a histopathologist for adequacy. No immediate complications were found (arrows).

The patient is a 45-year-old female with SCD, with stroke as a complication and hypothyroidism. Her medications and transfusion history included hydroxyurea, thyroxine, and folic acid and chronic transfusion for stroke prevention in SCD. She presented to the outpatient department (OPD) with a three-month history of mild dysphagia to solids and progressive anterior neck swelling, which started as an accidental finding in a routine ultrasound. She set appointments for her hypothyroidism at the OPD (Figure 1).

**Figure 2:**
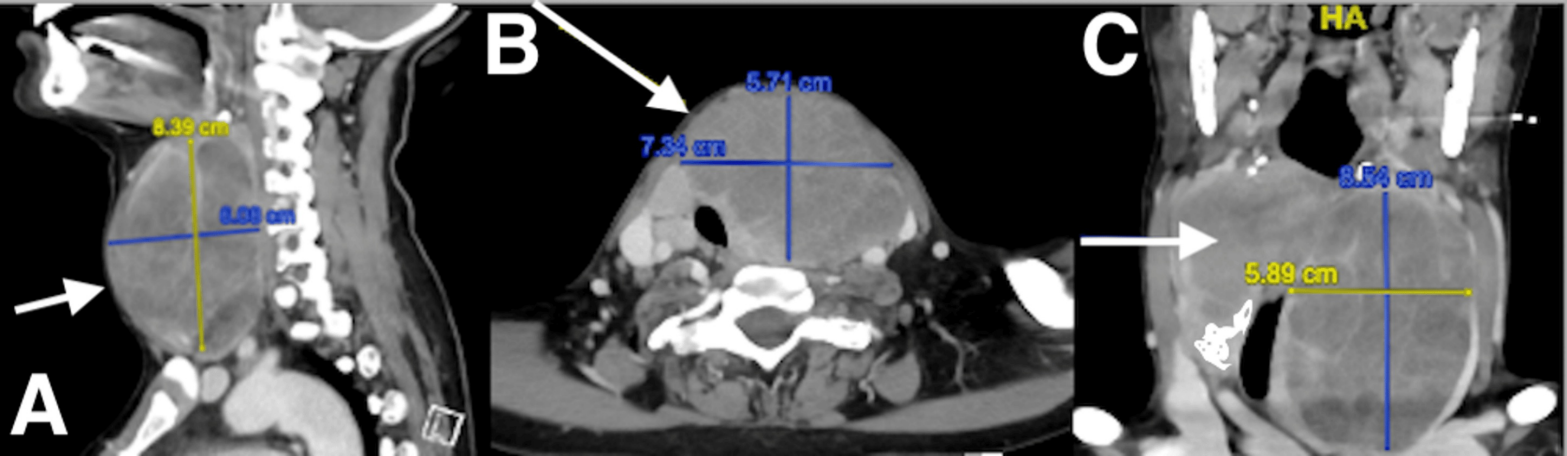
First computed tomography (CT) of the thyroid (A) Sagittal, (B) transverse horizontal, and (C) coronal dimensions on computed tomography (CT) of the anatomical thyroid bed pre-total thyroidectomy (arrows).

The first neck ultrasound revealed enlarged thyroid lobes and isthmus, showing a heterogeneous echopattern and multiple variable-sized nodules (the largest, in the right lobe, measures 0.66 x 1.2 cm; a left lobe nodule is a large, solid, heterogeneous nodule with internal hyperechoic nodules). A Doppler study with the Thyroid Imaging Reporting and Data System (TIRADS), which is the universally accepted modality for assessment and evaluation of thyroid nodules, showed increased gland vascularity. Sonographic features of multi-nodular goiter, that is, RT lobe hypoechoic nodule = TIRADS IV and right lobe with peripheral calcification TIRADS IV-V, were both highly suspicious for malignancy according to TIRADS; the left lobe large nodule = TIRADS II was not suspicious; and hyperechoic module = TIRADS III was mildly suspicious. FNA cytological assessment of the largest accessible nodule from the left thyroid nodule demonstrated cellular smears showing a predominantly polymorphic population of lymphocytes. A few clusters of oncocytic cells are visible. The cell block showed reactive lymphoid cells and rare oncocytic cells. Cytological features were consistent with Hashimoto's thyroiditis, without cytologic atypia or architectural features suggestive of malignancy. Three months later, the patient experienced rapid thyroid enlargement with increasing compressive symptoms in the form of orthopnea and dysphagia, which is considered a red flag in the presence of an expansive neck mass, which initiated and escalated a more extensive therapeutic course. She was reevaluated by neck CT, showing bilateral massive enlargement of the thyroid lobes with heterogeneous enhancement, and tracheal deviation (Figure 2).

The increasing mass effect and suspicious radiologic features encouraged surgical referral, which concluded with total thyroidectomy.

**Figure 3:**
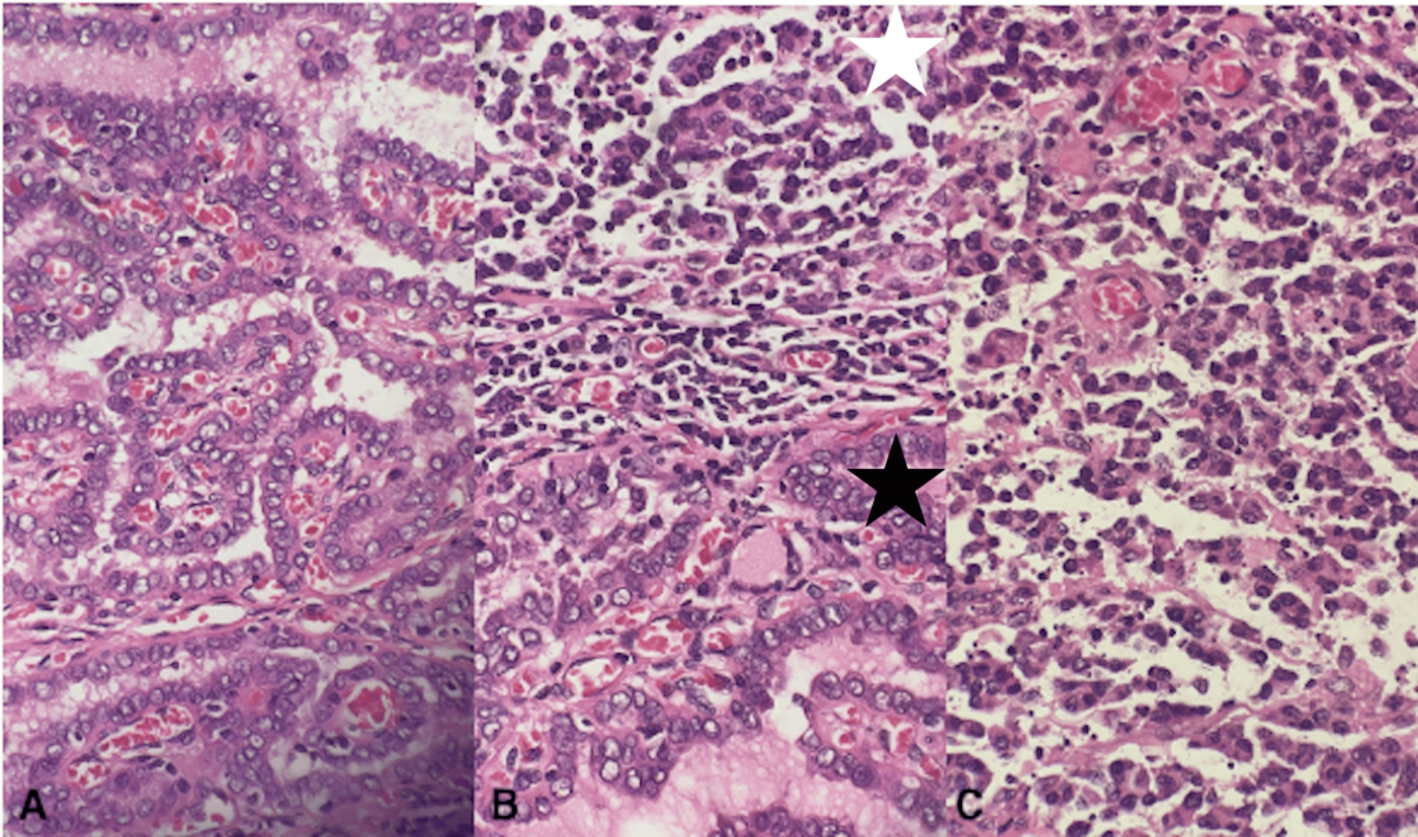
Histopathological examination of post thyroidectomy tissue (A) Papillary thyroid carcinoma (PTC): papillary configurations lined by enlarged follicular cells with nuclear overlapping, clearing, and grooves. (B) Coexistence of high-grade B cell lymphoma in the upper part (white star) with PTC in the lower part at the same microscopic field (black star). (C) High-grade B cell lymphoma: intermediate- to large-sized malignant cells with round to irregular nuclei, vesicular chromatin, and moderate amount of cytoplasm, with frequent apoptotic bodies.

Macroscopic examination of the total thyroidectomy specimen revealed that both lobes are enlarged with multiple tan soft homogenous unencapsulated nodules occupying the whole lobes on cut sectioning. Microscopic examination revealed diffuse proliferation of intermediate to large-sized malignant discohesive cells with round to irregular nuclei, vesicular chromatin, distinct nucleoli, and a moderate amount of cytoplasm. Frequent mitoses, apoptotic bodies, and areas of necrosis are present. These cells were confirmed to be malignant lymphoid B cells by immunohistochemistry studies, as they show diffuse strong positivity for CD45, CD20, CD79a, CD10, and BCL6 with high-grade features based on the proliferative index of Ki-67 (90%). Further, diffuse coexpression of CD10 and BCLe suggests a follicular center cell derivation. In addition, in the left lobe, there is a focus (1 mm) of papillary thyroid microcarcinoma showing papillary configurations lined by enlarged follicular cells with nuclear overlapping, clearing, and grooves, confined to the thyroid parenchyma without extra-thyroidal extension or vascular invasion (Figure 3).

**Figure 4:**
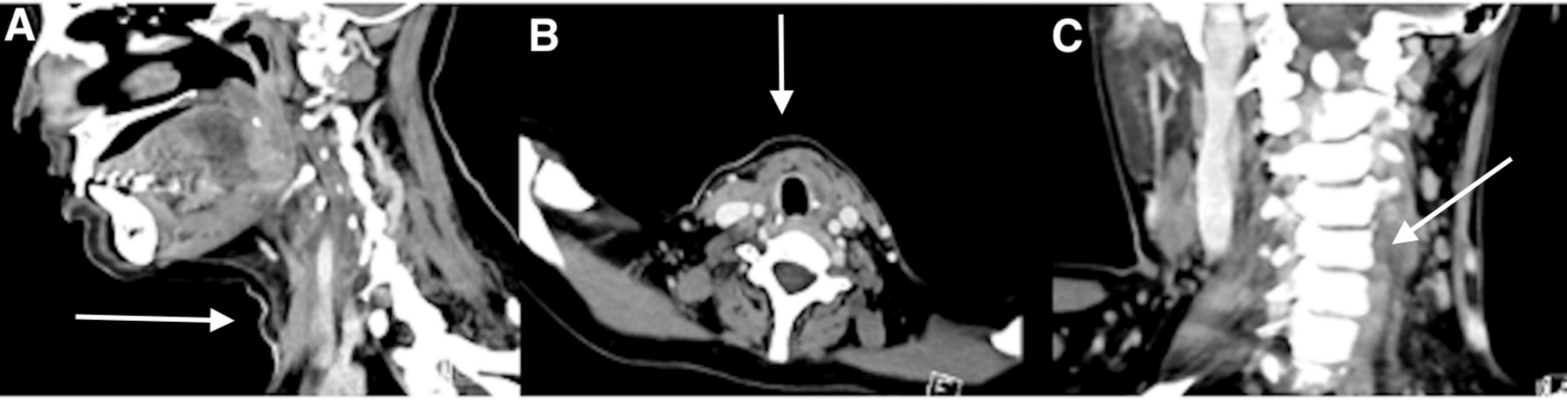
Second computed tomography (CT) of the thyroid post-resection and completion of chemotherapy (A) Sagittal, (B) transverse horizontal, and (C) coronal dimensions on computed tomography (CT) of the anatomical thyroid bed post total thyroidectomy and completion of chemotherapy (arrows).

18F-FDG PET-CT demonstrated intense uptake in the thyroid bed and two small, mildly avid bilateral cervical lymph nodes, without evidence of distant disease. No CNS involvement was found for lumbar puncture and brain and spine magnetic resonance. According to the Ann Arbor classification, the disease was Stage IIE. The patient completed six cycles of R-CHOP (rituximab, cyclophosphamide, doxorubicin, vincristine, and prednisone) chemotherapy. She endured therapy well, with manageable hematologic toxicity and complications. Follow-up CT of the neck demonstrated complete resolution of the compressive thyroidal bed with concurrent clinical improvement of dysphagia and orthopnea (Figure 4).

**Figure 5:**
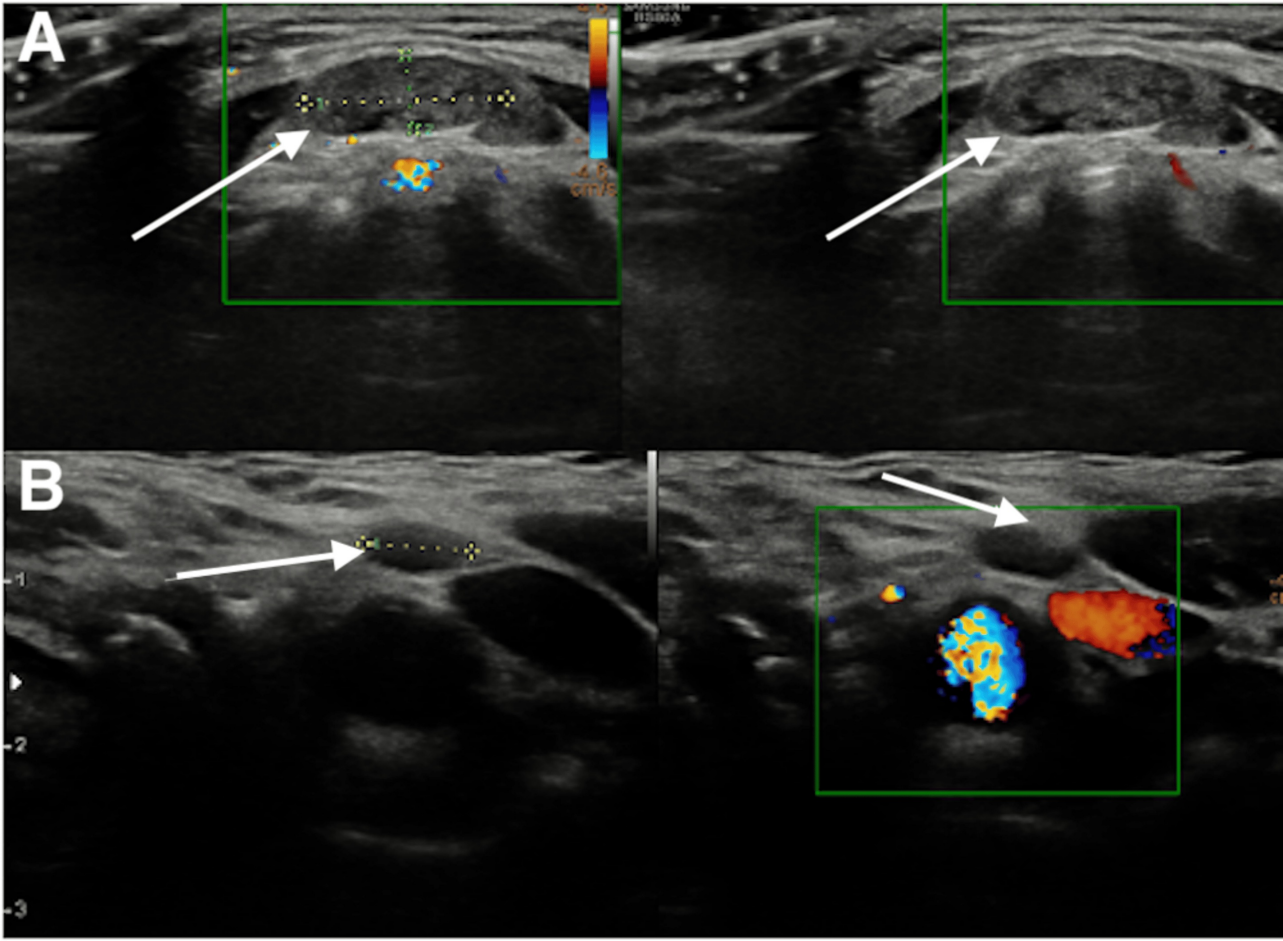
This neck ultrasound shows post treatment lymphadenopathy (A) Multiple mid-line supra-thyroid oval-shaped soft tissue lesions. (B) Left cervical lymphonodes bilateral cervical non-specific LN enlargement noted. Clear thyroid bed, showing minimal residual thyroid tissue. No localized collection, normal parotid and submandibular glands, and normal neck vasculature. Histopathological assessment confirmed the benign presence (arrows).

**Figure 6:**
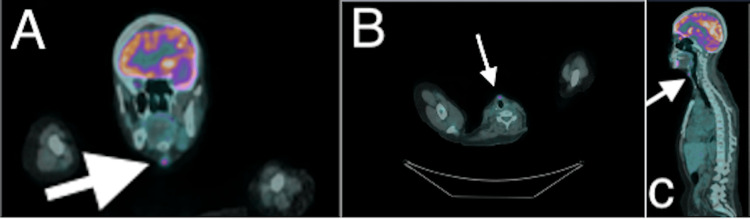
PET scan post resection and rituximab, cyclophosphamide, doxorubicin, vincristine, and prednisone (R-CHOP) (A) Transverse horizontal, (B) coronal, and C) sagittal section midline FDG-avid lesion measuring 1.2 × 0.8 cm anterior to the cricothyroid cartilage initially mistaken for recurrence. istopathological assessment revealed a benign nature.

The final post-surgical resection and chemotherapeutic agents PET-CT scan displayed complete resolution of uptake at the original sites. However, the PET-CT revealed a new, raised suspicion for relapse. Repeated neck ultrasound demonstrated a small, hypoechoic solitary lesion near the thyroidectomy anatomical bed (Figure 5). Given the prospect of relapse, a core needle biopsy was performed, skipping the FNA for a better diagnostic yield, which was encouraged by the current literature, the first initial encounter, and the history of malignancy the patient has had. A detailed histopathologic examination of the biopsy revealed benign reactive lymphoid tissue and a thyroid remnant, without evidence of malignancy. There was no recurrence of lymphoma or carcinoma, and the patient has remained clinically in remission. She maintained surveillance follow-up by hematology, oncology, endocrinology, and surgery. Figure 6 presents the PET scan post resection and R-CHOP.

Summary of the case

A 45-year-old case of SCA with hypothyroidism initially presented during follow-up OPD with endocrine and US revealed suspicious nodules. FNA was done on the nodule and was inconclusive. Patient continued surveillance in OPD.

After three months, the patient revisited OPD with exaggerated compressive symptoms and was referred to endocrine surgery for total thyroidectomy.

Histopathological assessment revealed the presence of dual malignancies, PTC and PTL. The patient was then referred to hematology, and she received complete cycles of R-CHOP.

A post-treatment PET scan showed a small suspicious area of uptake initially mistaken for recurrence. Final histopathological assessment revealed a benign lesion, and the patient is currently in remission with serial monitoring in OPD.

## Discussion

The present case exhibited diagnostic challenges, particularly due to the initially inconclusive (FNA) results, which revealed cytological features representative of Hashimoto's thyroiditis without evidence of malignancy. Our case highlights a critical limitation of FNA in the setting of underlying autoimmune thyroiditis, where lymphocytic infiltration can obscure malignant cells, especially in early or heterogeneous lesions. Despite the benign FNA, the patient's thyroid nodule progressed rapidly in size over a short period, accompanied by compressive symptoms, prompting surgical intervention. Despite its central role, FNA has limited sensitivity in diagnosing PTL, especially in the setting of lymphocytic thyroiditis. The presence of dense lymphoid infiltrates in Hashimoto's thyroiditis can obscure malignant lymphoid populations. In this case, the FNA misclassified lymphoma as Hashimoto's disease, delaying diagnosis. Clinical suspicion and judgment should dominate the management approach, remaining high when thyroid enlargement progresses rapidly or compressive symptoms start, even in the presence of benign cytology. The incidental finding of papillary thyroid microcarcinoma in this patient highlights the importance of comprehensive histopathological assessment, even after an initial assessment has identified a potential culprit. While PTL and PTC are individually rare, their coexistence is even more exceptional and may reflect a shared inflammatory or autoimmune heritage [7].

Post-treatment imaging modalities are important for thyroid malignancy surveillance; however, FDG uptake in PET-CT is not specific to malignancy. Regarding PET-CT, post-thyroid cancer treatment, physiological, inflammatory changes, and granulomatous reactions can all yield inaccurate results. In the present case, the PET-CT scan demonstrated a questionable lesion, which subsequent histopathological assessment confirmed to be benign lymphoid hyperplasia. Thus, these false-positive PET-CT results could be present in the post-treatment setting, especially when inflammatory or reactive processes are present.

In our case, the post-surgical inflammatory activity contributed to the PET avidity, underscoring the necessity of biopsy before initiating further therapy based only on imaging. An article reported that the use of rituximab can cause increased PET-CT signal uptake, which the current case received. Rituximab has been used in combination with hydroxyurea in the context of SCD and Hashimoto's disease, which has encouraged further research in the area of positive PET-CT signals [8]. Our examination stresses the significance of histopathological assurance before anticipating regression or relapse, especially in patients with prior neck surgery or inflammatory conditions. The current study underlines that Hashimoto's thyroiditis might disguise the cytological characteristics of lymphoma on FNA. In addition, clinical progression, rapid goiter growth, compressive symptoms, and systemic features indicate the need for escalated diagnostics rather than benign cytology. Post-treatment FDG PET-CT positivity in the absence of suggestive symptoms or biopsy-proven presence of malignancy may not represent relapse. A multidisciplinary discussion and approach are crucial to avoid misclassification and overtreatment. Utilizing non-invasive yet informative modalities, especially ultrasound, in patients with chronic inflammatory thyroid diseases, such as Hashimoto's thyroiditis, and implementing the TIRADS system for screening, are a few of the most effective tools to help identify who is at higher risk and who needs an accelerated and escalated approach [9,10].

## Conclusions

This case of primary high-grade thyroid lymphoma with coincidental papillary microcarcinoma in an SCD-affected patient highlights the diagnostic sophistication presented by inconclusive FNA, prompt tumor advancement, and misleading imaging. Clinical wariness and tissue verification remain paramount, notably if post-treatment imaging implies recurrence. The case highlights the importance of multidisciplinary evaluation, integrated imaging, and comprehensive histological examination, as well as personalized therapy, in complex oncological cases. Continuous supervision remains necessary due to the danger of secondary complications and recurrence. Constant screening with US among Hashimoto's thyroiditis as a potential risk factor is critical for early identification of any malignant transformation and better prognostic value.
